# Optimization of deep eutectic solvents-based ultrasonic-assisted three-phase partitioning extraction of polysaccharides from *Pleurotus tuber-regium* and its anti-ulcerative colitis activity

**DOI:** 10.1016/j.ultsonch.2026.107780

**Published:** 2026-02-13

**Authors:** Ge Dong, Xinyu Song, Chang Cai, Jiaheng Chen, Xuedong Dai, Fangfei Guo, Tongcun Zhang, Qian Zhang

**Affiliations:** a Institute of Biology and Medicine, College of Life and Health Sciences, Wuhan University of Science and Technology, Wuhan 430081, China; b School of Pharmacy, Guangdong Pharmaceutical University, Guangzhou 510006, China; c Saifu Laboratories Suzhou Co., Ltd., Jiangsu 215123, China; d Guangzhou Institutes of Biomedicine and Health, Chinese Academy of Sciences, Guangzhou 510530, China

**Keywords:** Polysaccharides, Deep eutectic solvent-based ultrasonic-assisted three-phase partitioning, Ulcerative colitis, *Drosophila*

## Abstract

Based on the various anti-inflammatory and antioxidant bioactivities of *Pleurotus tuber-regium* and its polysaccharides (PTPs), they represent promising candidates for complementary or alternative therapeutic strategies against Ulcerative Colitis (UC), but conventional extraction methods are often inefficient or environmentally unfriendly. This study presents a novel integrated deep eutectic solvents (DESs)-based ultrasonic-assisted three-phase partitioning (TPP) extraction technique of PTPs to solve the dual challenges of inefficient traditional extraction methods and the unexplored therapeutic potential of PTPs against UC. First, it establishes an optimized and sustainable extraction process, enhancing the yield and potential bioavailability of PTPs. Single-factor assays followed by Box-Behnken Design (BBD) were employed to optimize key parameters: DES-2 (choline chloride:1,6-hexanediol = 1:2, molar ratio), temperature 40℃, material-liquid ratio 1:33 mg/mL, ammonium sulphate mass fraction 15%, upper and lower phase (ammonium sulphate: DES-2) volume ratio 1:2, time 40 min, power 320 W. Structural characterization utilized UV spectrophotometer (UV–Vis) and Fourier transform infrared (FT-IR) spectrometer, high-performance gel permeation chromatography (HPGPC), and scanning electron microscopy (SEM). Monosaccharide composition was analyzed via PMP derivatization and HPLC. Second, it provides the first comprehensive preclinical evidence, using dextran sulfate sodium (DSS)-induced *Drosophila* colitis model, to systematically validate the anti-UC efficacy of the optimized PTPs extract. The findings bridge a critical gap, offering both an advanced extraction method for efficient utilization of PTPs and a pharmacological foundation for developing PTPs as a promising nutraceutical or botanical drug candidate for inflammatory bowel disease management.

## 1 Introduction

Ulcerative colitis (UC) is an inflammatory, chronic, idiopathic bowel disease with complex etiology [1]. Patients with UC suffered with bloody diarrhoea, abdominal pain, and tenesmus, leading to impaired quality of life, disability, and even colorectal cancer [2], [3]. Its worldwide incidence and prevalence have been steadily rising, which leads to a significant global health burden. The general approach for maintenance treatment for UC involves of biologic agents (e.g., anti-TNFα antibodies, vedolizumab, and ustekinumab), aminosalicylates, corticosteroids, and immunomodulators [4], [5]. However, these maintenance treatments have significant limitations including limited efficacy, immunogenicity, primary non-response or loss of response over time, and the risk of serious adverse effects including infections and malignancy [5]. Consequently, some patients still require surgery, and there is an urgent need for exploring safer, more cost-effective, and novel therapeutic agents. In this context, natural products, especially bioactive polysaccharides derived from medicinal fungi [6], [7], have gained increasing attention due to their immunomodulatory [8], anti-inflammatory, and gut-barrier protective properties [9] with generally favorable safety profiles, as promising candidates for the development of complementary or alternative strategies against UC.

Building upon the promise of natural polysaccharides for UC treatment, *Pleurotus tuber-regium* (Fr.) Sing., an edible and medicinal mushroom traditionally played important parts of Asia and Africa for its health-promoting properties, presents itself as a promising candidate [10]. Numerous studies have documented the bioactivities associated with *P. tuber-regium* polysaccharides (PTPs) [11], [12], including potent antioxidant effects through free radical scavenging [11], [13], significant anti-inflammatory properties [14], as well as immunomodulatory [15] and gut microbiota-regulating potentials [16]. Such a multi-target bioactivity profile is highly relevant to the complex pathogenesis of UC [7], [9], [17].

However, a significant research gap persists: the specific efficacy of *P. tuber-regium* polysaccharides against UC remain largely unexplored and lack systematic validation. Furthermore, to fully use its therapeutic potential, a sustainable method for extracting bioactive polysaccharides are needed to be developed to optimal utilization of *P. tuber-regium,* as conventional extraction techniques often suffer from drawbacks such as low yield, long extraction times, high energy consumption, and the use of large volumes of potentially hazardous organic solvents [18].

Conventional methods for fungal polysaccharide extraction, such as hot water (HWE), microwave (MAE), acid/alkaline, and enzyme-assisted extraction, are often faced significant limitations: high energy/equipment consumption, prolonged processing times and reduces efficiency, which may compromise the structural integrity and bioactivity of the target compounds [19], [20]. To further streamline and optimize extraction method, in this study, deep eutectic solvents (DESs)-based ultrasonic-assisted three-phase partitioning (TPP) extraction was used and presented a promising green strategy with superior efficacy.

In three-phase partitioning (TPP) system, crude biological extracts can be induced phase separation through the sequential or simultaneous addition of a water-miscible organic solvent, typically *tert*-butanol (t-butanol), and an inorganic salt, most commonly (NH_4_)_2_SO_4_ [21], [22], [23], [24]. The TPP system has been widely used to isolate and purify a variety of biologically active molecules, including proteins, enzymes, oils, and polysaccharides [25], [26]. TPP has many advantages over conventional extraction and separation techniques, such as a simple, time efficient, economical, but it also has disadvantages. A major limitation of conventional TPP is its reliance on t-butanol as the primary organic solvent [27]. T-butanol exhibits several undesirable properties: volatility and flammability, toxicity and environmental impact. Therefore, the development of alternative, greener solvents to replace t-butanol in TPP systems represents a significant research imperative within green chemistry and sustainable bioprocessing [28].

DESs exhibit markedly depressed melting points while offering exceptional tunability of physicochemical properties and can be engineered to selectively solubilize target polysaccharides [29], [30] to improve extraction efficiency. Most components (e.g., choline chloride, glycerol) of DESs are bio-based, low-toxicity, and biodegradable [31], also are recyclability to reduces waste [32]. Crucially, polysaccharides extracted by DES retain bioactivity due to mild processing conditions, avoiding chain scission or functional group alterations common in harsh extractions [33]. When combined with advanced TPP, DESs can substitute for t-butanol to reduce the disadvantages [28].

In summary, to advance an efficient, low-damage extraction process for *P. tuber-regium* polysaccharides (PTPs) and proof of concept on its anti-ulcerative colitis bioactivity, this study established a synergistic DES-based ultrasonic-assisted TPP method for the extraction and purification of PTPs with more efficient*.* Extraction parameters were optimized through single-factor experiments followed by response surface methodology (RSM) employing a Box-Behnken design (BBD). The extracted polysaccharide fraction (designated as PTP-DES2) underwent comprehensive structural characterization, including molecular weight distribution analysis, monosaccharide compositional profiling, and Fourier-transform infrared (FT-IR) spectroscopy. Finally, the well-established dextran sulfate sodium (DSS)-induced *Drosophila* model of ulcerative colitis was employed to assess the in vivo therapeutic efficacy.

## 2 Materials and methods

### 2.1 Materials and Reagents

*P. tuber-regium* were obtained from Anguorui Shenyuan Chinese Herbal Medicine Co., Ltd. and identified by the Drug Identification Department of the School of Traditional Chinese Medicine, Guangdong Pharmaceutical University. The *Drosophila* strains used in this study, including w^1118^ and Esg-Gal4, UAS-GFP/Cyo, were obtained from the Qidong Fangjing Biotechnology Co., Ltd. (Jiangsu, China). Bovine Serum Albumin (BSA) was purchased from Biosharp Life Sciences (Biosharp, China). Triton X-100 was sourced from GBCBIO Technologies Inc. (Guangzhou, China). All chemicals for the experiments were of analytical grade and obtained from local vendors.

### 2.2 Production of standard curve

The glucose standard curve was generated using a modified phenol sulfuric acid method [24]. Briefly, a stock solution (3  mg/mL) was prepared from anhydrous D glucose. Serial dilutions yielded six concentrations (0.02344–0.75  mg/mL). Each standard (30  μL) was analyzed in triplicate by adding 30  μL of 5% phenol and 160  μL concentrated sulfuric acid. After 30  min incubation at room temperature, absorbance was read at 490  nm. The curve was plotted as absorbance versus glucose concentration.

### 2.3 Extraction and determination of polysaccharides from *p. Tuber-regium*

The extraction and determination of *P. tuber-regium* were performed using a three-phase partitioning system combined with ultrasound-assisted extraction, following a modified previously method [25], [27]. Briefly, Polysaccharides were extracted using an ultrasound-assisted aqueous three-phase partitioning system. Briefly, 0.05 g of powdered sample was mixed with a DES and ammonium sulfate solution at optimized solid/liquid ratio, salt concentration, and phase ratio, followed by ultrasonication. After centrifugation, polysaccharides in the bottom phase were precipitated with 95% ethanol to 80% final concentration, stored at 4 °C for 12 h, and collected by centrifugation at 4000 r/min for 5 min. The precipitate was redissolved in distilled water, and lyophilized to obtain crude polysaccharides.

The polysaccharide content was quantified by the phenol–sulfuric acid method. A sample solution was reacted with 5% phenol and concentrated sulfuric acid, and absorbance was measured at 490 nm. The content was calculated using a glucose standard curve. The polysaccharide yield was calculated according to the following formula:

$$=\frac{C\times V\times n}{M\times 1000}\times 100\%$$

In this formula: C: Concentration, mg/mL; V: Volume, mL; n: Dilution fold; M: Mass of *P. tuber-regium*.

### 2.4 Screening of DESs

The screening of DESs was conducted according to a previously reported method with modifications [29], [30]. DESs were prepared as listed in Table 1 by mixing the components at specified molar ratios, followed by heating at 80 °C with stirring until a homogeneous liquid formed. The polysaccharide yield, defined as the amount of crude polysaccharide extracted from *P. tuber-regium* powder, was used as the key performance indicator. The DES formulation that yielded the highest amount of polysaccharides was selected for all subsequent extraction experiments.

**Table 1:** DESs with different compositions.

**No.**	**Hydrogen bond acceptor- Hydrogen bond donor**	**Molar ratio**
DES-1	Choline chloride-Isopropanol	1:2
DES-2	Choline chloride-1,6-Hexanediol	1:2
DES-3	Choline chloride-Glycerol	1:2

### 2.5 Single-Factor experiments

Polysaccharide yield was used as the response to optimize six extraction parameters. Each factor was tested individually while keeping the other conditions constant (unless stated otherwise, the default conditions were: extraction time 40  min, temperature 40 °C, ultrasonic power 320  W, material/liquid ratio 1:20  mg/mL, ammonium sulfate 15%, phase ratio 1:2). The tested ranges were:

• extraction time: 20, 30, 40, 50, 60  min
• temperature: 30, 40, 50, 60, 70 °C
• ultrasonic power: 240, 280, 320, 360, 400  W
• material/liquid ratio: 1:10, 1:20, 1:30, 1:40, 1:50  mg/mL
• ammonium sulfate mass fraction: 10, 15, 20, 25, 30%
• upper/lower phase volume ratio (aqueous salt: DES): 1:0.5, 1:1, 1:1.5, 1:2, 1:2.5

All experiments were performed in triplicate on 0.05  g samples following the procedure described in Section 2.3.

### 2.6 Response surface methodology optimization experiments

Based on the results of the single factor experiments, a three-factor, three-level RSM design was implemented using BBD in Design-Expert software (Version 13.0). This design generated a total of 17 experimental runs. Each run was performed according to the prescribed conditions, and the polysaccharide yield was determined. The resulting data were analyzed to determine the optimal extraction parameters. The optimal process conditions were subsequently validated experimentally. Furthermore, the efficiency of the optimized method was compared against conventional extraction techniques, including hot water extraction (HWE), ultrasonic-assisted water extraction (UAWE), and a system where *tert*-butyl alcohol was substituted for the DES.

### 2.7 Determination of protein content of *p. Tuber-regium*

Protein content was determined using the Bradford assay with BSA as the standard. A calibration curve was prepared using BSA solutions (0–100  µg/mL). Samples (1  mg/mL) and standards were mixed with Coomassie Brilliant Blue G-250 reagent, and absorbance was measured at 592  nm. The protein content in polysaccharides extracted by the optimized method, as well as in those obtained by HWE, UAWE, and the tert–butanol system, was calculated from the standard curve.

### 2.8 Structural characterization

#### 2.8.1 Ultraviolet spectral analysis

UV–vis spectral analysis was performed on the PTP-DES2. The sample was prepared at a concentration of 1.0 mg/mL, and the absorbance was scanned from 200 to 400 nm.

#### 2.8.2 Infrared spectral analysis

An aliquot of the PTP-DES2 (2 mg/mL) was mixed with potassium bromide (KBr), and pressed into a translucent pellet. FT-IR spectroscopy was subsequently performed with scanning conducted across the wavelength range of 4000 to 400 cm^−1^.

#### 2.8.3 The molecular weight analysis

The molecular weight of PTP-DES2 were investigated via high-performance gel permeation chromatography (HPGPC), using a TSK-GEL G-3000 PWXL gel column (TOSOH Bioscience, Yamaguchi, Japan) with RID-10A detector (Shimadzu, Japan). A 0.02 M KH_2_PO_4_ solution served as the mobile phase, flowing at a rate of 0.5 mL/min. Various T-series dextran standards with different molecular weights (T1000, T500, T70, T40, T10, and T5) were sequentially analyzed to construct the calibration curve, correlating retention time with the logarithm of the respective molecular weights (Supplementary Fig. S2). The molecular weight distribution of PTP-DES2 was calculated using the following formula:

y = 14.83865–1.30621x + 0.06122x^2^-0.00135x^3^, R^2^ = 0.9994.

#### 2.8.4 Analysis of monosaccharide composition

The sample (5 mg) was hydrolyzed with 2 M trifluoroacetic acid (TFA) at 120 °C for 4 h. After removal of TFA, the hydrolysate was derivatized with 1-phenyl-3-methyl-5-pyrazolone (PMP) in alkaline medium (70 °C, 30 min). Excess reagent was removed by chloroform extraction. Analysis was performed on an Agilent 1260 HPLC with a Shim-pack VP-ODS column (250 × 4.6 mm, 5 μm) and a mobile phase of phosphate buffer (pH 6.7)–acetonitrile (83:17, v/v) at 1.0 mL/min. Detection was at 250 nm. A mixed standard of ten monosaccharides (mannose, ribose, rhamnose, glucuronic acid, galacturonic acid, glucose, galactose, arabinose, xylose, and fucose) was processed and analyzed under the same conditions.

#### 2.8.5 Scanning electron microscopy (SEM) analysis

The morphological characteristics of PTP-DES2 were examined by SEM (Carl Zeiss GeminiSEM300, Oberkochen, Germany) and images were obtained at 200×, 500×, and 1000 × magnification.

### 2.9 Anti-Inflammatory activity

#### 2.9.1 *Drosophila* medium Preparation

Sucrose (94.86 g), calcium chloride (0.726 g), agar (10 g), and yeast (32.19 g) were dissolved in 636 mL of distilled water with continuous stirring to ensure homogeneity and prevent agglomeration. The solution was heated until viscous, then mixed with a cornmeal suspension (77.7 g in 250 mL water). The mixture was boiled with stirring, supplemented with propionic acid (1 mL), and removed from heat. The medium was dispensed into culture tubes (2–3 mL) or bottles (20–25 mL), allowed to solidify at room temperature, plugged, and stored at 4 °C [34].

#### 2.9.2 *Drosophila* survival test

*Drosophila melanogaster* (w^1118^) were collected 3–5 days post-eclosion and anesthetized with isoflurane. For each sex, 30 flies were placed into a culture tube (three replicate tubes per group) and starved for 2  h in empty tubes.

Flies were then transferred to assay tubes containing filter papers saturated with 200 μL of one of the following test solutions: 5% sucrose (control group, NC), 4% DSS in 5% sucrose (model group, DSS), or one of four concentrations (1%, 2%, 5%, 10%) of PTP-DES2 and 4% DSS in 5% sucrose (treatment groups). The filter papers and solutions were replaced every 24 h, and mortality was recorded daily for 7 d [35].

#### 2.9.3 *Drosophila* intestinal morphology Change test

Female *Drosophila melanogaster* (w1118) (4–5 days post-eclosion) were treated for 4 d with filter papers saturated with 200 μL of one of the following solutions: 5% sucrose (NC), 4% DSS in 5% sucrose (DSS), or 5% PTP-DES2, 4% DSS in 5% sucrose (PTP-DES2). After treatment, 10 to 15 fruit flies per group were necropsied. Their intestines were collected and mounted on microscope slides in a 70% glycerol solution and measured and analyzed under a microscopy [36]. The experiment was performed in triplicate.

#### 2.9.4 Quantification of intestinal stem and progenitor cells in *Drosophila*

Female esg-Gal4; UAS-GFP/CyO flies (4–5 d post‑eclosion) expressed green fluorescent protein were treated for 4 d with solutions of 5% sucrose (NC), 4% DSS in 5% sucrose (DSS), or 4% DSS plus 5% PTP‑DES2; filter papers and solutions were replaced daily. Ten to fifteen intestines per group were necropsied, fixed in 4% paraformaldehyde (PFA) for 20  min, washed with PBT (phosphate-buffered saline with 0.1% Triton X-100), and stained with 4′,6-diamidino-2-phenylindole (DAPI) for 10  min. Samples were mounted in 70% glycerol and imaged by fluorescence microscopy to visualize GFP‑labeled stem/progenitor cells [36].

#### 2.9.5 Analysis of cell death in the *Drosophila* intestine by 7-AAD staining

Female *Drosophila melanogaster* (w^1118^) (4–5 d post‑eclosion) were treated for 4 d with 5% sucrose (NC), 4% DSS in 5% sucrose (DSS), or 4% DSS plus 5% PTP‑DES2; solutions were replaced daily. Intestines (10–15 per group) were dissected and stained with 5  μg/mL 7-Aminoactinomycin D (7‑AAD) in the dark for 30  min. After washing with PBT, tissues were fixed in 4% PFA for 30  min, washed again, counterstained with DAPI for 10  min, and mounted in 70% glycerol for fluorescence imaging [37].

### 2.10 Statistical analysis

Data was reported as means ± SD, and all extractions and fly trials were conducted with a minimum of three replicates. Group variances were analyzed using one-way ANOVA with multiple comparisons in GraphPad Prism 10. Statistical significance was set at *P* < 0.05 for ANOVA results, with significance levels indicated as *P < 0.05, **P < 0.01, ***P < 0.001.

## 3 Results and Discussion

### 3.1 Screening of deep eutectic solvents

A standard curve was established to quantify glucose concentration, demonstrating a strong linear relationship between concentration and absorbance (Supplementary Fig. S1). The linear regression equation was defined as y = 4.48347C + 0.22787, with a coefficient of determination (R^2^) of 0.9985, confirming excellent model fit.

**Fig. 1:**
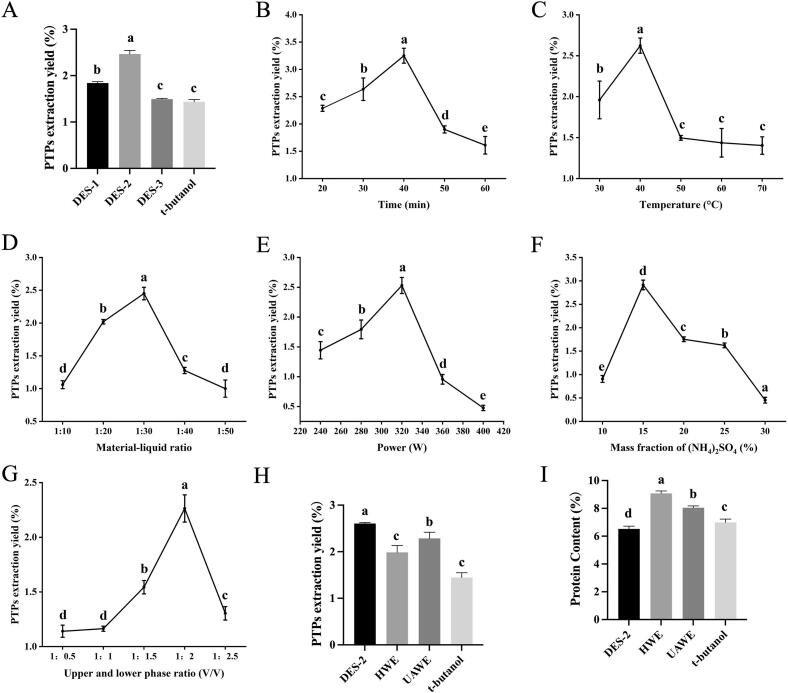
Screen of DESs, single-factor experiments and validation experiments for DES-TPP. (A) Effect of different DESs, (B) extraction time, (C) extraction temperature, (D) material-liquid ratio, (E) ultrasonic power, (F) mass fraction of (NH_4_)_2_SO_4_, and (G) upper and lower phase ratio on PTPs extraction yield. (H) different extraction methods on PTPs extraction yield, (I) different extraction methods on protein content of PTPs. Data are expressed as the mean ± SD (n = 3). Different letters indicate the statistical significance at *P* < 0.05.

The extraction efficiency of DESs, formulated with choline chloride as the hydrogen bond acceptor and various hydrogen bond donors at specific molar ratios, varied significantly (Fig. 1A). Among the tested formulations and t-butanol, DES-2, composed of choline chloride and 1,6-hexanediol as the hydrogen bond donor, demonstrated the highest extraction yield at 2.47%. Consequently, DES-2 was selected as the hydrogen bond donor for all subsequent experiments.

### 3.2 Extraction efficiency analysis

#### 3.2.1 Effect of extraction time

The extraction yield of polysaccharides was significantly influenced by time (Fig. 1B). The yield increased with prolonged extraction duration, reaching a maximum at 40 min. This initial increase is likely attributable to enhanced cell wall disruption and improved polysaccharide dissolution facilitated by extended ultrasonic exposure. However, beyond this optimum point, a further increase in time resulted in a decline in yield, potentially due to the co-extraction of impurities. Therefore, 40 min was selected as the optimal extraction time for all subsequent experiments.

#### 3.2.2 Effect of extraction temperature

The extraction temperature exerted a significant influence on the polysaccharide yield from *P. tuber-regium* (Fig. 1C). The yield increased with rising temperature up to an optimum of 40 °C, beyond which it decreased and subsequently plateaued. The initial increase in yield can be attributed to enhanced molecular kinetic energy and improved solubility of the polysaccharides at elevated temperatures. The observed decline beyond the optimum temperature is likely a consequence of the thermal degradation of the polysaccharide structures. Based on these results, 40 °C was selected as the optimal temperature for subsequent extractions.

#### 3.2.3 Effect of Material-to-Liquid ratio

As shown in Fig. 1D, the extraction rate reached its peak at a material-to-liquid ratio of 1:30 mg/mL. However, with a further increase in the ratio, the polysaccharide extraction rate began to decline gradually.

A low solvent volume (high material-to-liquid ratio) prevents the complete dissolution of polysaccharides, resulting in a low extraction rate. When the solvent volume is excessive (low material-to-liquid ratio), the solubility of polysaccharides no longer increases. Instead, the excessive solvent leads to the dissolution of additional soluble components, which reduces the relative concentration and extraction efficiency of the polysaccharides, thereby decreasing the observed extraction rate. Therefore, a material-to-liquid ratio of 1:30 mg/mL was selected for subsequent experiments.

#### 3.2.4 Effect of ultrasonic power

As shown in Fig. 1E, the polysaccharide extraction rate initially increased with rising ultrasonic power, reaching a maximum at 320 W, beyond which it gradually decreased. This enhancement is primarily attributed to the higher energy input at increased power levels, which promotes cell wall disruption and improves the dispersion of *P. tuber-regium* powder within the solvent system, thereby accelerating polysaccharide dissolution. However, excessively high ultrasonic power may degrade the polysaccharide structures and activity, leading to a reduction in extraction yield. Based on these findings, an ultrasonic power of 320 W was selected for all subsequent experiments.

#### 3.2.5 Effect of ammonium sulfate mass fraction

As shown in Fig. 1F, the extraction yield of *P. tuber-regium* polysaccharides initially increased and then decreased with increasing ammonium sulfate ((NH_4_)_2_SO_4_) mass fraction, reaching a maximum at a concentration of 15%. This trend occurs because a higher (NH_4_)_2_SO_4_ mass fraction stabilizes interactions between macromolecules and enhances the stability of the three-phase system, thereby promoting the dissolution of polysaccharides into the aqueous phase. However, when the (NH_4_)_2_SO_4_ mass fraction exceeds the optimum, a strong salting-out effect occurs. This reduces the availability of water to solubilize the *P. tuber-regium* powder, causing more material to partition into the upper or middle phases of the three-phase system rather than the target aqueous phase. Therefore, an ammonium sulfate solution with a mass fraction of 15% was selected for all subsequent experiments.

#### 3.2.6 Effect of upper − lower phase volume ratio

As shown in Fig. 1G, the polysaccharide extraction yield initially increased and subsequently decreased with an increasing volume ratio of the upper phase to the lower phase, reaching a maximum at a ratio of 1:2. This pattern can be attributed to the role of DES in enhancing the buoyancy of proteins and other macromolecules, facilitating their separation from the aqueous phase, while simultaneously dissolving impurities such as lipids and pigments. When the volume of DES is insufficient, it fails to synergize fully with the (NH_4_)_2_SO_4_ solution, leading to inadequate protein precipitation and a suboptimal polysaccharide extraction yield. Conversely, an excessive amount of DES increases the viscosity of the solution, which reduces the molecular migration rate and consequently diminishes the polysaccharide extraction efficiency. Therefore, a volume ratio of 1:2 (upper phase: lower phase) was selected as the optimal condition for subsequent experiments.

### 3.3 Response surface test results and analysis

Based on the single-factor experiments, the material-to-liquid ratio (A), the mass fraction of ammonium sulfate (B), and the upper-lower phase volume ratio (C) were selected as critical factors. The polysaccharide extraction yield was designated as the response value for the response surface optimization.

The factors and their corresponding levels for the response surface optimization design are presented in Table 2. The experimental design, based on the Box-Behnken model, and the corresponding results are summarized in Table 3.

**Table 2:** Response surface factors and levels.

**Factors**	**Level**		
	**−1**	**0**	**1**
A: Material ratio-Liquid (mg/mL)	20	30	40
B: Ammonium sulfate mass fraction (%)	10	15	20
C: The upper-lower phase volume ratio (mL/mL)	1.5	2	2.5

**Table 3:** Box-Behnken response surface design and corresponding response values.

**Run**	**Material-liquid ratio (g/mL)**	**Ammonium sulfate mass fraction (%)**	**The upper-lower phase volume ratio (mL/mL)**	**Extraction yield (%)**
1	1:20	20	1:2	1.29
2	1:30	15	1:2	2.78
3	1:30	15	1:2	2.74
4	1:20	15	1:2.5	0.92
5	1:20	15	1:1.5	1.46
6	1:30	15	1:2	2.63
7	1:40	20	1:2	1.08
8	1:30	15	1:2	2.73
9	1:30	10	1:2.5	0.63
10	1:30	20	1:1.5	1.37
11	1:40	10	1:2	1.33
12	1:30	15	1:2	3.15
13	1:30	10	1:1.5	0.92
14	1:30	20	1:2.5	1.28
15	1:40	15	1:2.5	2.55
16	1:40	15	1:1.5	1.97
17	1:20	10	1:2	0.98

The results from the 17 test points, designed using the BBD, were subjected to statistical analysis using Design-Expert 13 software. A regression analysis of the experimental data yielded a second-order polynomial equation describing the relationship between the polysaccharide extraction yield (Y) and the independent variables: material-to-liquid ratio (A), ammonium sulfate mass fraction (B), and upper and lower phase volume ratio (ammonium sulfate: DES) (C). The equation is as follows:

Y = 2.81 + 0.2850A + 0.1450B − 0.0425C − 0.1400AB + 0.2800AC + 0.0500BC − 0.4805A^2^ − 1.16B^2^ − 0.6005C^2^.

The coefficient of determination (R^2^) for this model was 0.9253, indicating a satisfactory fit to the experimental data.

The analysis of variance, goodness-of-fit and adequacy of the models were summarized in Table 4. The regression model for the polysaccharide extraction yield of *P. tuber-regium* was statistically significant (*P* < 0.05), while the lack-of-fit term was not significant (*P* > 0.05), indicating the model is well-suited for predicting the response. Based on the F-values, the order of influence of each factor on the extraction yield was as follows: material-to-liquid ratio > ammonium sulfate mass fraction > upper and lower phase volume ratio.

**Table 4:** Regression model analysis of variance.

**Source**	**Sum of Squares**	**df**	**Mean Square**	**F- value**	**p-value**	
Model	10.12	9	1.12	9.63	0.0035	significant
A − Material ratio-Liquid	0.6498	1	0.6498	5.56	0.0504	
B − Ammonium sulfate mass fraction	0.1682	1	0.1682	1.44	0.2691	
C − The upper-lower phase volume ratio	0.0144	1	0.0144	0.1238	0.7354	
AB	0.0784	1	0.0784	0.6714	0.4396	
AC	0.3136	1	0.3136	2.69	0.1453	
BC	0.01	1	0.01	0.0856	0.7783	
A^2^	0.9721	1	0.9721	8.33	0.0235	
B^2^	5.62	1	5.62	48.15	0.0002	
C^2^	1.52	1	1.52	13	0.0087	
Residual	0.8174	7	0.1168			
Lack of Fit	0.6573	3	0.2191	5.47	0.0671	not significant
Pure Error	0.1601	4	0.04			

**Fig. 2:**
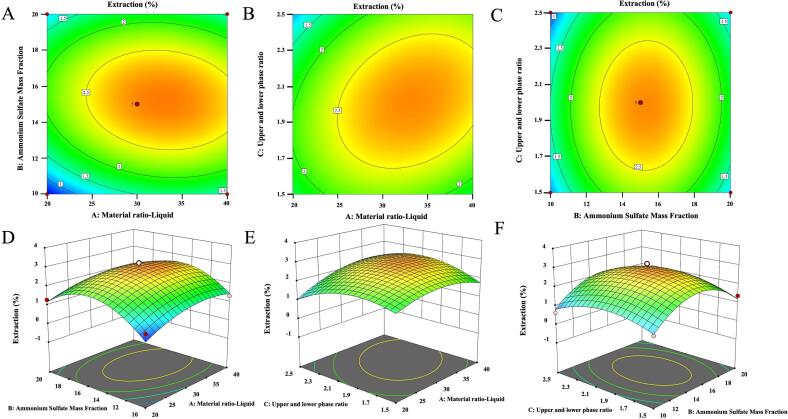
Response surface and contour plots. (A and D) the effect of the interaction between ammonium sulphate mass fraction and water content of DES; (B and E) ammonium sulphate mass fraction and the ratio of DES to crude extract; (C and F) water content of DES and the ratio of DES to crude extract on the extraction yield of PTPs.

Response surface and contour plots (Fig. 2) were generated and analyzed using Design-Expert 13 software to visualize the changes and interactive trends of the response value under the influence of multiple factors.

### 3.4 Determination of optimal process conditions and validation

Response surface analysis yielded the following optimal process conditions: a material-to-liquid ratio of 1:32.999 mg/mL, an ammonium sulfate mass fraction of 15.225%, and an upper-lower phase volume ratio (ammonium sulfate: DES) of 1:2.019, with a predicted extraction yield of 2.85%. For practical operational convenience, the parameters were adjusted to material-to-liquid ratio of 1:33 mg/mL, ammonium sulfate mass fraction of 15%, and upper and lower phase volume ratio of 1:2. Validation experiments conducted under these adjusted conditions resulted in an actual polysaccharide yield of 2.61% (Fig. 1H), which was close to the predicted value, demonstrating the model's good predictive capability.

For comparative purposes, several alternative extraction methods — hot water extraction, ultrasonic water extraction, and a method using *tert*-butanol instead of DES were also performed under the same optimized physical conditions (ratio, mass fraction, phase ratio). The polysaccharide yields for these methods were 1.99%, 2.29%, and 1.44%, respectively. These results confirm that the DES-based extraction method developed in this study provides a superior yield compared to the conventional techniques tested, validating the effectiveness of the optimization process.

### 3.5 Determination of protein content in *p. Tuber-regium* polysaccharide extract

A standard curve for protein concentration was established, demonstrating a strong linear relationship between concentration and absorbance (Supplementary Fig. S3). The linear regression equation was calculated as y = 0.3346x + 0.1771, with a coefficient of determination (R^2^) of 0.9926, indicating an excellent fit. As shown in Fig. 1I, the protein content of PTP-DES2 was 6.51%. For comparison, the protein yields obtained from *P. tuber-regium* using hot water extraction, ultrasonic water extraction, and the method employing *tert*-butanol instead of the DES were 9.08%, 8.04%, and 6.99%, respectively.

### 3.6 Structural characterization

#### 3.6.1 Results of UV spectral analysis

**Fig. 3:**
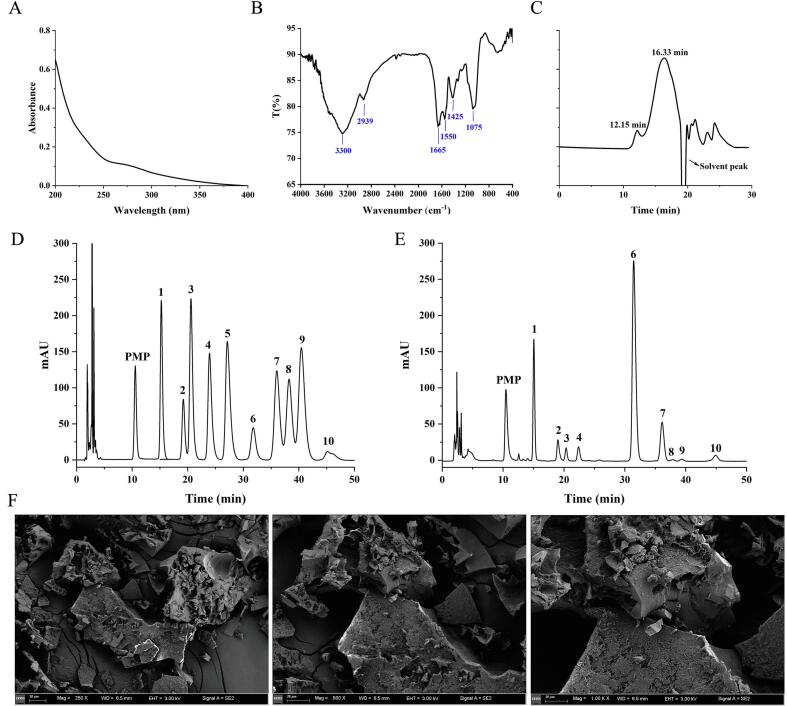
Primary characterization of PTP-DES2. (A) The UV spectrum of PTP-DES2, (B) The IR spectrum of PTP-DES2, (C) HPGPC spectrum of PTP-DES2, (D) PMP-HPLC of ten standard monosaccharides (1, mannose; 2, ribose; 3, rhamnose; 4, glucuronic acid; 5, galacturonic acid; 6, glucose; 7, galactose; 8, xylose; 9, arabinose; 10, fucose), (E) PMP-HPLC of hydrolyzed PTP-DES2, (F) SEM images of PTP-DES2. × 200, ×500, and × 1000 magnification.

The UV–Vis spectrum (Fig. 3A) exhibited a slight absorption peak at 272 nm, indicating the presence of a small quantity of protein within the PTP-DES2. This result is consistent with the protein content of 6.55% determined in Section 3.5**.** Additionally, a characteristic terminal absorption peak for polysaccharides was observed at 200 nm.

#### 3.6.2 Results of FT-IR spectroscopy analysis

The FT-IR spectrum of PTP-DES2 is shown in Fig. 3B. Characteristic broad absorption peaks were observed at approximately 3300 cm^−1^ and 2939 cm^−1^, corresponding to O-H stretching vibrations and C-H stretching vibrations of –CH_2_ groups, respectively. These are hallmark features of polysaccharide compounds.

Additional diagnostic peaks were identified: an absorption at 1665 cm^−1^ is associated with C=O stretching, confirming the presence of saccharides; the peak at 1550 cm^−1^ is attributed to the asymmetric stretching vibration of free carboxyl groups (–COO^-^); the absorption at 1425 cm^−1^ arises from C-H bending vibrations; and the band at 1075 cm^−1^ is assigned to the asymmetric stretching vibration of the ether bond (C-O-C) in the pyranose ring. Collectively, these spectral features confirm the presence of typical sugar components within the PTP-DES2.

#### 3.6.3 The molecular weight analysis

The results from the HPGPC spectrum revealed that PTP-DES2 is a partially purified polysaccharide (Fig. 3C and D), and the molecular weight of PTP-DES2 is approximately from 9.01 kDa to 383.94 KDa based on the calibration curve in Supplementary Fig. S2.

#### 3.6.4 Monosaccharide composition analysis

As shown in Fig. 3E, PTP-DES2 was composed of mannose, ribose, rhamnose, glucuronic acid, glucose, galactose, xylose, arabinose, and fucose. The molar ratios of these monosaccharides were determined to be 230.01: 38.77: 23.40: 25.41: 379.38: 72.46: 1.00: 1.80: 9.27.

Glucose, mannose, and galactose were the predominant monosaccharides. Glucose exhibited the highest peak area and the greatest molar proportion, suggesting that the *P. tuber-regium* may possess a glucose-dominated backbone structure.

#### 3.6.5 SEM analysis

The morphological structure of PTP-DES2 was determined using SEM. As shown in Fig. 3F, PTP-DES2 displays a fragmented structure with debris of various sized. The surfaces of these fragments appear rough, with noticeable cracks and voids, indicating a porous and brittle characteristic. When magnified, the fragments show irregular shapes, and the surface texture is more pronounced, with small particles and uneven protrusions. Furthermore, the surfaces of PTP-DES2 are characterized by numerous tiny pores and uneven textures, and the edges of the fragments are sharp, reflecting the complex and heterogeneous nature of the polysaccharide at a fine scale.

### 3.7 In vivo Anti-Ulcerative colitis evaluation

#### 3.7.1 Effect of PTP-DES2 on the survival of *Drosophila melanogaster*

**Fig. 4:**
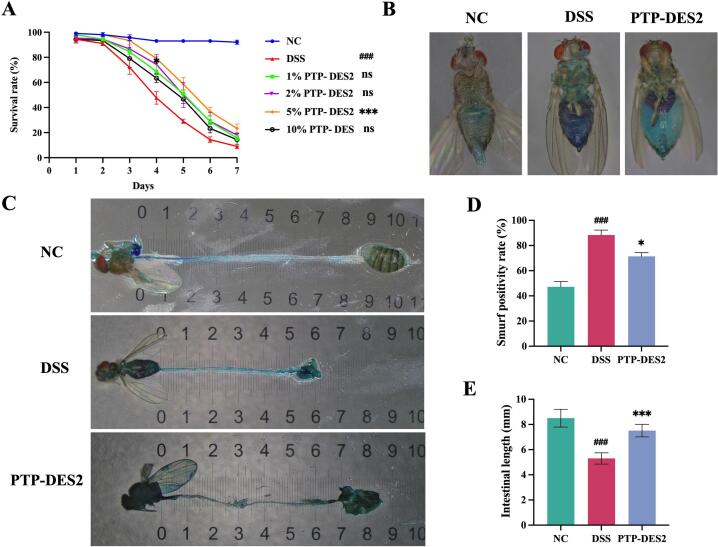
Effects of PTP-DES2 on survival, intestinal barrier and intestinal length in UC *Drosophila*. (A) Effect of PTP-DES2 on UC *Drosophila* survival, (B) Representative images of flies in different group after feeding blue dye, (C) Representative experiments of intestine length, (D) Quantification of the Smurf-positive rate (number of Smurf-positive flies/total number of flies, n = 3), (E) Quantitative analysis of the intestinal length (n = 8). *P < 0.05, ^***^P < 0.001 *vs.* the DSS group, ^###^*P* < 0.001 *vs.* the NC group.

As shown in Fig. 4A, the survival rate of *Drosophila melanogaster* in the control group remained at a high level without significant fluctuation. In contrast, the survival rates in the groups treated with PTP-DES2 were as follows: 15.67% for the 1% group, 18.00% for the 2% group, 23.33% for the 5% group, and 14.33% for the 10% group. All treatment groups exhibited higher survival rates compared to the model group.

Among the four dosage groups, the 5% PTP-DES2 group demonstrated the highest survival rate. Consequently, 5% concentration was selected for all subsequent experiments.

#### 3.7.2 Effects of PTP-DES2 on on the intestinal permeability

DSS, due to its sulfate groups carrying high negative charges, has toxic effects on colonic epithelium, which can induce tissue erosion and ultimately compromise barrier integrity, resulting in increased intestinal permeability. The degree of blue dye infiltration in Drosophila after staining can be used to assess the integrity of the intestinal barrier, thus studying the effects of PTP-DES2 on intestinal permeability. As shown in Fig. 4B and D, Drosophila in the DSS group exhibited blue coloration throughout their bodies. The blue dye solution ingested by the NC group only infiltrated the mouthparts and digestive tract, while the infiltration levels of PTP-DES2 group was intermediate between the NC and DSS groups. The Smurf-positive rate in the DSS group was 88.4%, increased by 41.2% compared to the NC group, suggesting that DSS successfully induced an intestinal or physiological abnormality model in flies. Compared to the DSS group, the Smurf positive rate in the PTP-DES2 group was decreased by 17.1%, which significantly alleviated DSS-induced intestinal leakage. Thus, it can be concluded that PTP-DES2 have significant effects on improving intestinal permeability.

#### 3.7.3 Effects of PTP-DES2 on *Drosophila* intestinal morphology

As shown in Fig. 4C and E, the intestines of *Drosophila* in the control group exhibited the greatest length and displayed partially folded ends. In contrast, intestinal length was significantly shortened in the model group. Treatment with PTP-DES2 resulted in a notable increase in intestinal length compared to the model group, although it remained slightly shorter than that observed in the control group.

These results indicate that PTP-DES2 significantly ameliorates DSS-induced intestinal atrophy in *Drosophila*, effectively improving and maintaining intestinal morphology and enhancing the ability to resist external stimuli.

#### 3.7.4 Effect of PTP-DES2 on the number of intestinal stem cells and enterocytes of *Drosophila*

**Fig. 5:**
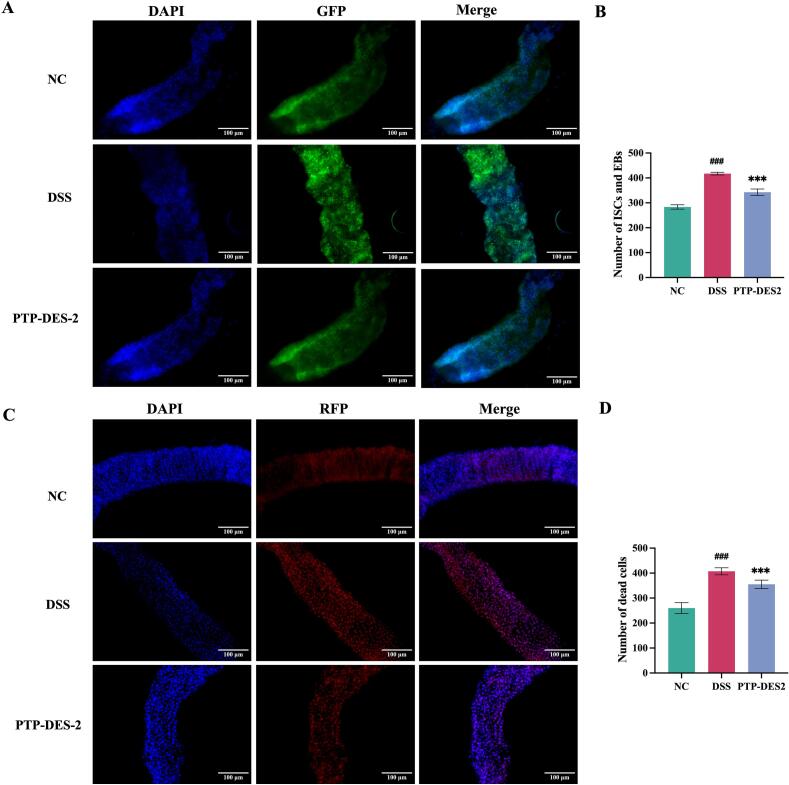
PTP-DES2 protects *Drosophila* from DSS-induced over-proliferation of ISCs and EBs, IECs death. (A) Intestinal Esg-GFP staining, (B) Number of ISCs and EBs (n = 8), (C) Intestinal 7-AAD staining, (B) Number of dead IECs (n = 8). ^***^P < 0.001 *vs.* the DSS group, ^###^*P* < 0.001 *vs.* the NC group.

As shown in Fig. 5A and B, GFP-positive cells (visualized as green fluorescent dots) were least numerous in the control group. The model group exhibited the highest number of GFP-positive cells within the intestinal tract. Treatment with PTP-DES2 reduced the quantity of these cells compared to the model group, though the count remained higher than that in the control group.

These results suggest that PTP-DES2 effectively mitigates DSS-induced over proliferation of intestinal stem and progenitor cells in *Drosophila*, albeit with a limited regulatory capacity. This indicates a certain degree of amelioration of inflammation-induced intestinal damage by the polysaccharide treatment [38].

### 3.8 Effect of PTP-DES2 on *Drosophila* intestinal epithelial cell death

As shown in Fig. 5C and D, the number of intestinal epithelial cell deaths was lowest in the control group and highest in the model group. Treatment with PTP-DES2 resulted in an intermediate level of cell death, which was significantly lower than that in the model group but higher than that in the control group.

These results demonstrate that PTP-DES2significantly inhibits the extensive DSS-induced death of intestinal epithelial cells in *Drosophila*. This supports the hypothesis that the polysaccharide exerts an ameliorative effect on inflammation-related intestinal damage [38].

## 4 Conclusion

This study successfully developed and optimized a DES-based ultrasonic-assisted TPP method for the efficient extraction of polysaccharides from *P. tuber-regium*. The optimized protocol demonstrated an extraction yield of 2.61% and a product purity characterized by a protein content of 6.51%, outperforming conventional hot water, ultrasonic water, and *tert*-butanol-based extraction methods. These results highlight the potential of the DES system as an efficient and selective medium for extracting bioactive polysaccharides.

Structural characterization analyses confirmed that the obtained polysaccharide (PTP-DES2), which has a molecular weight ranging from 9.01 to 383.94 KDa, exhibits characteristic structural features of polysaccharides and is composed of various monosaccharides, including mannose, ribose, rhamnose, glucuronic acid, glucose, galactose, xylose, arabinose, and fucose. More importantly, *in vivo* evaluation revealed significant anti-inflammatory and intestinal-protective effects of PTP-DES2 in a *Drosophila melanogaster* model of ulcerative colitis. Treatment with 5% PTP-DES2 led to marked improvements in intestinal morphology, suppressed excessive proliferation of intestinal stem cells and enterocytes, and reduced epithelial cell death.

In summary, this work not only establishes a green and efficient extraction process but also systematically demonstrates the significant intestinal anti-inflammatory activity of the specific polysaccharide fraction obtained through this optimized method for the first time. These findings provide a solid experimental foundation for developing *P. tuber-regium* polysaccharides as potential therapeutic agents or functional food ingredients for intestinal health.

## CRediT authorship contribution statement

**Ge Dong:** Writing – original draft, Validation, Project administration, Methodology, Formal analysis. **Xinyu Song:** Validation, Software, Methodology, Data curation. **Chang Cai:** Methodology, Investigation, Data curation. **Jiaheng Chen:** Visualization, Validation. **Xuedong Dai:** Validation, Project administration. **Fangfei Guo:** Visualization, Data curation. **Tongcun Zhang:** Writing – review & editing, Supervision. **Qian Zhang:** Writing – review & editing, Supervision, Project administration, Funding acquisition, Conceptualization.

## Declaration of competing interest

The authors declare that they have no known competing financial interests or personal relationships that could have appeared to influence the work reported in this paper.
