# Supplementary material for: Optimization of deep eutectic solvents-based ultrasonic-assisted three-phase partitioning extraction of polysaccharides from Pleurotus tuber-regium and its anti-ulcerative colitis activity
Source: Ultrason Sonochem. 2026 Feb 13;127:107780. doi: 10.1016/j.ultsonch.2026.107780 (PMC12925531; doi:10.1016/j.ultsonch.2026.107780)
Supplement: Supplementary Data 1 [file mmc1.docx]

**Supplementary Figures**

**
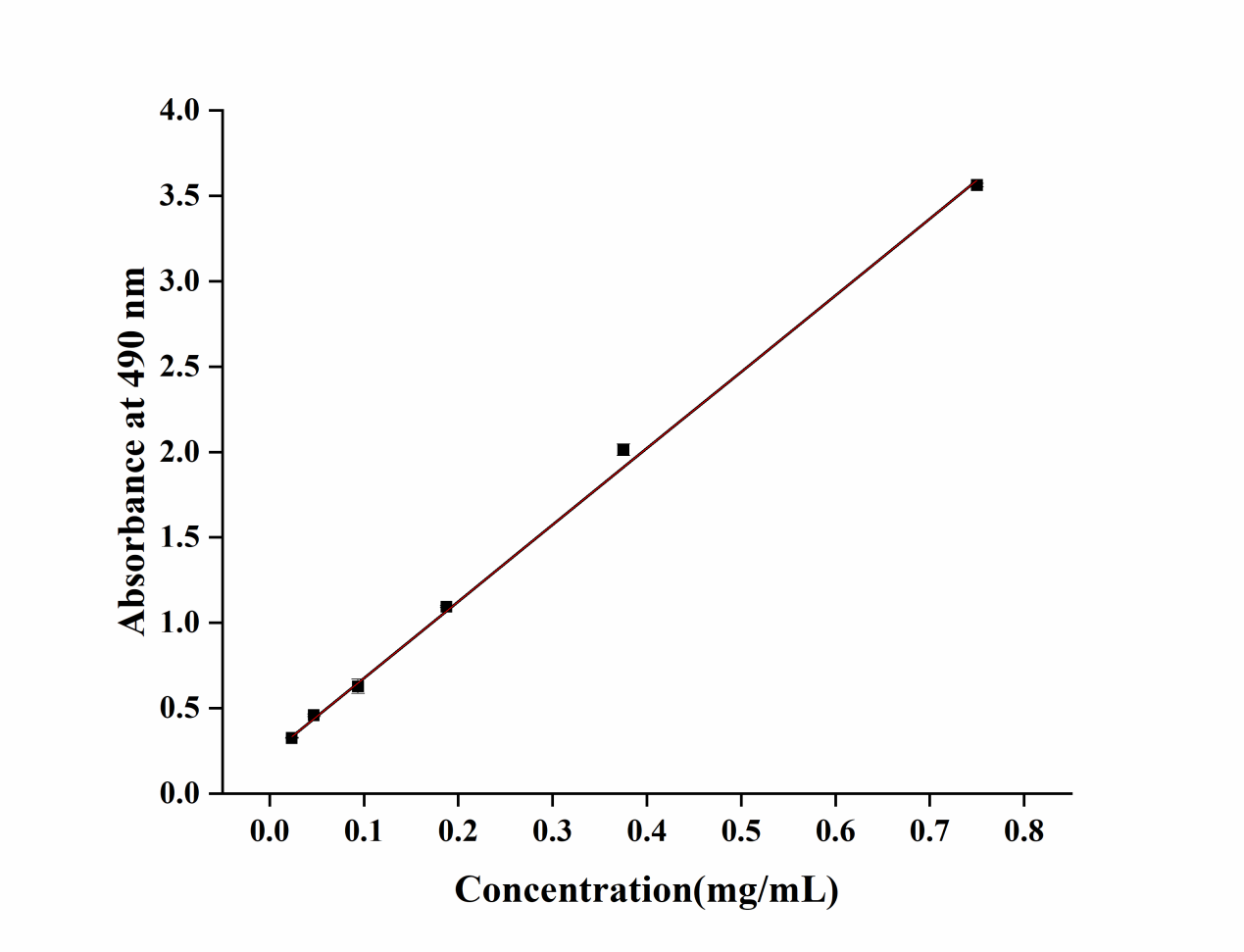
**

Fig. S1 The calibration curve of total sugar.


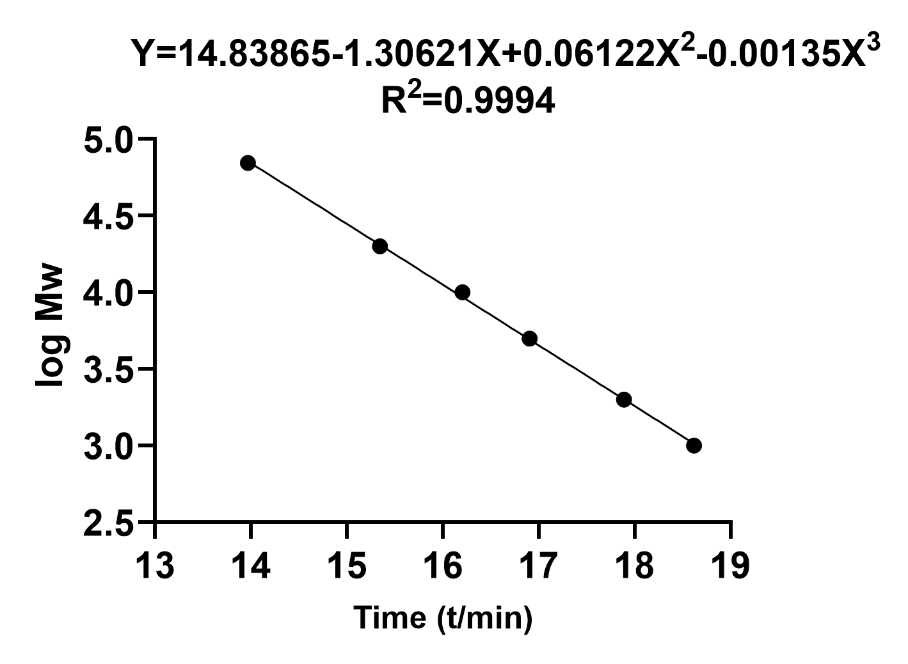


Fig. S2 The calibration curve of molecular weight.





Fig. S3 The calibration curve of protein content.
